# Comparison of the clinical efficacy of different acupuncture methods in the treatment of post-stroke central facial paralysis—a network meta-analysis based on randomized controlled trials

**DOI:** 10.3389/fneur.2026.1790069

**Published:** 2026-03-27

**Authors:** Juanshu Cao, Hang Xing, Wenlong Hu, Qiuxia Xu, Li-yan Cui, Liyu Hu, Xiaomei Zhang, Shengyong Bao

**Affiliations:** Department of Rehabilitation Medicine, Shenzhen People’s Hospital (The First Affiliated Hospital, Southern University of Science and Technology; The Second Clinical Medical College, Jinan University), Shenzhen, China

**Keywords:** acupuncture, central facial paralysis, comparative effectiveness, network meta-analysis, randomized controlled trial, stroke

## Abstract

**Background:**

Post-stroke central facial paralysis (CFP) is a common post-stroke complication that affects the quality of life. The current conventional treatment has limitations in efficacy and safety. As a traditional medical therapy, acupuncture and moxibustion have shown potential in the treatment of facial paralysis, but the differences in efficacy of different acupuncture methods are still lacking a systematic comparison.

**Objective:**

Compare the efficacy differences of different acupuncture methods or combined methods in treating CFP with acupuncture alone.

**Methods:**

The system retrieved randomized controlled trials (RCTs) published in Chinese- and English-language databases through 1 January 2026. The eligible literature was screened. The risk of bias was evaluated using the RoB2 tool, and a network meta-analysis was conducted using the random-effects model within the framework of frequency analysis. The primary outcome indicators included total effective rate (TER) and facial disability index (FDI). Due to the different efficacy assessment criteria used in the original studies, the TER analysis was stratified according to the House–Brackmann (HB) grading standard and the “Chinese Traditional Medicine Disease Syndrome Diagnosis and Therapeutic Effect Standards” (Traditional Chinese Medicine [TCM]). The efficacy of the intervention measures was ranked using the Surface Under the Cumulative Ranking (SUCRA) curve value, and the quality of evidence was evaluated using the Confidence in Network Meta-Analysis (CINeMA) tool.

**Results:**

A total of 22 RCTs were included, involving 1,888 patients and 8 types of acupuncture intervention measures. Based on the HB criteria, 9 studies showed that acupuncture combined with conventional treatment (AandCT, SUCRA = 0.84), acupuncture combined with traditional Chinese medicine (AandTCM, SUCRA = 0.73), and penetrating acupuncture combined with moxibustion (PNTandMT, SUCRA = 0.68) ranked the top in improving TER. Based on the TCM criteria, 10 studies showed that fire needle (FN, SUCRA = 0.82), acupuncture combined with traditional Chinese medicine (AandTCM, SUCRA = 0.79), and scalp acupuncture (SA, SUCRA = 0.78) ranked the top. Regarding FDI improvement, 15 studies showed that botulinum toxin type A combined with acupuncture (BTTAandA, SUCRA = 0.90), cupping combined with acupuncture (CandA, SUCRA = 0.81), and the penetrating needling technique combined with moxibustion (PNTandMT, SUCRA = 0.79) had the best efficacy. The heterogeneity test showed low heterogeneity across the studies, and no significant publication bias was found in the analysis. Evidence quality evaluation showed that approximately 70% of the evidence was of moderate or higher quality.

**Conclusion:**

Different acupuncture therapies have their own advantages in improving central facial palsy after stroke: based on the HB criteria, acupuncture combined with conventional treatment or traditional Chinese medicine has better effects; based on the TCM criteria, fire needle and head acupuncture perform well; in terms of improving patients’ subjective function, botulinum toxin type A combined with acupuncture has a prominent advantage. The selection of acupuncture points centers on Hegu, Dicang, and Jiaqu, etc. The results of this study support acupuncture as a safe and cost-effective complementary therapy for clinical use, but future studies should further standardize the system for evaluating efficacy and conduct high-quality research to verify its long-term efficacy.

## 1 Introduction

According to the global stroke registry data, about 30–50% of stroke patients will have varying degrees of facial muscle dysfunction, among which central facial palsy (CFP) is dominant, which is closely related to ischemic injury of the cortical nuclear tract or the posterior limb of the internal capsule (1). In terms of demographic characteristics, facial paralysis following stroke is associated with age. Studies have shown that the incidence of facial paralysis in 50 stroke patients increases with age, and is accompanied by significant temporomandibular joint dysfunction (*p* < 0.05) (2). Mild facial paralysis can recover spontaneously, whereas moderate-to-severe facial paralysis is often accompanied by salivation, dysarthria, and masticatory weakness, which not only affect appearance but also reduce patients’ quality of life (3). More importantly, CFP was confirmed to be an independent predictor of stroke mortality (HR = 1.67), and its presence significantly increased the risk of death in patients by 67% (4). China’s Jiangsu area studies have found that in 526 cases of patients with acute ischemic stroke, facial paralysis is one of the nine key factors influencing the intravenous thrombolysis decision (5), one of which reflects the facial paralysis clinical weights in the evaluation system of stroke.

At present, the clinical treatment of CFP after stroke mainly relies on drug intervention and physical rehabilitation, but these conventional therapies still have obvious shortcomings in terms of efficacy and safety. Corticosteroid, as a first-line drug, can reduce neuroinflammatory response, but its long-term use may induce systemic adverse reactions such as hypertension, abnormal blood glucose, and osteoporosis (6). In terms of physical therapy, facial muscle electrical stimulation and functional training are widely used, but studies have shown that about 47% of patients still have moderate or severe facial muscle dysfunction in long-term follow-up, and treatment compliance is often significantly reduced due to the long course of treatment (usually 6–12 months). What is more noteworthy is that traditional therapies for special types of facial paralysis caused by brainstem infarction often have little effect (7). In recent years, new neuromodulation techniques, such as transcranial magnetic stimulation (TMS), have shown some potential, but there remains a lack of high-quality evidence for their use in post-stroke facial paralysis. Although neurosurgical interventions such as microvascular decompression are effective for specific causes, the postoperative complication rate of approximately 5.3% limits their widespread use (8). These therapeutic dilemmas highlight the limitations of existing therapies: the single target of drug action, the slow onset of physical therapy, and the uncertain risk–benefit ratio of invasive procedures. Therefore, there is an urgent need to explore safer and effective alternative therapies.

In the face of the limitations of standard therapies in the treatment of post-stroke CFP, it is particularly important to explore complementary and alternative therapies. As a representative treatment of traditional medicine, acupuncture and moxibustion have shown unique value in the field of nervous system diseases in recent years. Meta-analysis showed that acupuncture was significantly better than conventional medicine and exercise rehabilitation therapy in improving House–Brackmann (HB) facial nerve grading system scores and clinical efficacy, with good safety (9). From a mechanistic perspective, acupuncture may play a role through multiple pathways such as regulating neuroplasticity, improving local microcirculation, and reducing neuroinflammation (10). In terms of medical cost control, traditional Chinese medicine (TCM) characteristic therapy has been designated a national priority for development due to its standardized programs and cost-effectiveness. Economic analysis shows that in facial paralysis therapy, acupuncture and moxibustion treatment costs accounted for 48.05% of the total cost of traditional Chinese medicine, which is far lower than the conventional Western medicine treatment (11). However, there are still some controversies and methodological limitations in the treatment of post-stroke facial paralysis with acupuncture. The quality of existing studies is uneven, and the criteria for evaluating efficacy are not uniform. At present, most of them use the evaluation scale for peripheral facial paralysis and lack specific evaluation tools for CFP (12). Although acupuncture and moxibustion have been widely used in the clinical treatment of post-stroke facial paralysis, there is still a lack of systematic evaluation of the differences in efficacy between different acupuncture methods. The intervention methods, such as traditional acupuncture, electroacupuncture, and warming acupuncture, have their own characteristics. In clinical practice, they are often selected based on the doctor’s experience and lack of evidence-based support for the optimal choice. Therefore, conducting this study has multiple values: by integrating existing randomized controlled trial (RCT) data, the efficacy ranking of different acupuncture methods can be systematically evaluated; To improve the statistical power, especially suitable for the problem of insufficient sample size commonly found in acupuncture and moxibustion research; To identify the most cost-effective treatment plan and provide a reference for the optimal allocation of medical resources. The results of this study will provide a reliable basis for clinicians to make decisions and ultimately improve the prognosis of patients with post-stroke CFP.

## 2 Methods

### 2.1 Register

We strictly adhered to the Cochrane guidelines for reporting network meta-analysis (Preferred Reporting Items for Systematic Reviews and Meta-Analyses for Network Meta-Analyses [PRISMA-NMA]) and have prospectively registered this study.^1^

### 2.2 Inclusion criteria

Population: Met the diagnostic criteria for post-stroke central facial paralysis. Stroke: Stroke confirmed by head CT or MRI examination, or in accordance with the diagnostic criteria used in various studies, such as “Chinese Guidelines for the diagnosis and Treatment of Acute Ischemic Stroke” (13). Facial paralysis criteria: the diagnostic criteria used in the research species include facial expression muscle paralysis below the palpebral fissure on the opposite side of the lesion. When static, the nasolabial fold of the affected side became shallow, the angle of the mouth drooped or deviated, and the forehead wrinkles on both sides were symmetrical. The height of the eyebrow and the size of the palpebral fissure on both sides were equal. When dynamic, there is asymmetry on both sides of the nose, teeth, tympanic membrane, and cheeks; inability to whistle; and clinical manifestations such as salivation, food eating, and dysphagia; and normal bilateral eye closure and frowning (14).

Intervention measures: acupuncture methods (acupuncture, moxibustion, acupoint application, acupoint injection, etc.) were used alone or combined with other treatment methods (conventional treatment, cupping, traditional Chinese medicine, etc.). Frequency, period of treatment, and the specifications of the needles are not limited.

Control group: Acupuncture method or conventional treatment (such as drugs and conventional rehabilitation treatment). There were no restrictions on dosage, frequency, course of treatment, or specifications of needles.

Outcome indicators: (1) Total effective rate (TER); The facial nerve function was evaluated according to House–Brackmann (HB) classification (15). Cure: HBGS grade increased ≥3 or HBGS grade I; Marked efficacy: HBGS grade was increased by two grades compared with that before treatment; Effective: HBGS grade increased by one grade compared with that before treatment; Ineffective: there was no significant improvement in facial neuromuscular function and no change in HBGS grade. The clinical efficacy was evaluated according to the “Standards for the Diagnosis and Therapeutic Effect Evaluation of TCM Syndromes” (TCM) (16). If the facial paralysis symptoms disappeared, the facial function recovered, the corners of the mouth were not skewed, the eyelids could close completely, the facial muscles regained symmetry, it was considered as a complete recovery; if the facial paralysis symptoms basically disappeared and the facial function slightly recovered, the eyelids could close, the corners of the mouth skewed when smiling, the corners of the mouth were normal when the face was at rest, it was considered as a marked improvement; if the facial paralysis symptoms were relieved and the facial nerve function partially recovered, it was considered as effective; if none of the above standards were met, it was considered as ineffective. TER = the number of cured + the number of markedly effective + the number of effective/the total number of people × 100%. (2) Facial disability index (FDI) (17): FDI is a patient-rated scale consisting of 10 questions, divided into physical function subscale and social/well-being function subscale, using a 6-point rating scale (1 = severe disability, 6 = disability); The scores of each subscale were converted to 0–100, with 100 indicating disability.

Study type: Randomized controlled trial (RCT).

### 2.3 Exclusion criteria

Duplicate publications; articles that are not randomized controlled trials (reviews, animal trials, case reports, etc.) for which full text is not available; Data unavailable, missing, and unable to be analyzed; Outcome measures did not meet the inclusion criteria.

### 2.4 Literature search strategy

The combination of keywords, subject headings, and free words was used in China National Knowledge Infrastructure (CNKI), Wanfang database, VIP database, SinoMed, PubMed, Cochrane Library, Embase, and Web of Science Randomized controlled trials of acupuncture and moxibustion in the treatment of central facial paralysis after stroke published up to 1 January 2026 (Supplementary material – 1: Search strategy).

### 2.5 Literature screening and data extraction

The literature obtained from the database was imported into EndNote for deduplication, primary screening, and secondary screening to determine the literature included in the Meta-analysis. An Excel sheet was created to extract the basic data (first author, publication time, age, and course of disease) and core data (intervention plan, efficacy index, and treatment cycle) from the literature. More than two researchers were selected to complete the above process independently, and then they were checked with each other. If there was any disagreement, another researcher was invited to discuss and decide.

### 2.6 Risk of bias assessment of the included literature

RoB2 (18), a bias risk assessment tool recommended by the Cochrane Handbook, was used to evaluate the quality and risk of bias of the included literature. RoB2 was designed into five assessment domains: bias in the randomization process, bias due to deviation from the intended intervention, bias due to missing outcome data, bias due to outcome measurement, and bias due to selective reporting of results. The risk of bias in each domain was classified into three levels: low risk, unknown risk, and high risk. Two researchers to evaluate independently, comparing the results, such as disagreement, consulting a researcher at the third or discussing and making a decision.

### 2.7 Statistical analysis

Learning under the framework of this study adopts a frequency random-effects model for network meta-analysis. R version 4.4.0 was used for meta-analysis. Relative risk (RR) was used for binary variables, standardized mean difference (MD) for continuous variables, and the credibility interval (CI) was calculated. The *I*^2^ statistic test was used to determine the degree of heterogeneity. If *p* < 0.1 or *I*^2^ < 50%, there was no statistical heterogeneity. Statistical heterogeneity was indicated when *p* > 0.1 or *I*^2^ ≥ 50%, and sensitivity analysis was performed to explore the source of heterogeneity. The evidence network diagram of each outcome index was drawn. When a closed loop appeared, the inconsistency test was performed. If *p* > 005, the consistency model was used for analysis. If *p* < 005, the inconsistency will be reported, and the inconsistency will be checked by the node-splitting method. Will be ordered by the ending index, get the area under the curve (surface under the cumulative ranking, SUCRA). SUCRA was expressed as a percentage, with higher percentages indicating better interventions. When ending with 10 or more indicators in the literature, drawing a “comparison–correction” funnel figure, and Egger’s test to determine whether there is a publication bias and the possibility of a small sample effect exists. Meta-regression was used to explore the influence of different factors on the results. The Thompson–Sharp test and comparison–correction funnel plot were used to determine whether there was publication bias in the included studies.

### 2.8 Quality of evidence evaluation

We evaluated the quality of evidence using Confidence in Network Meta-Analysis (CINeMA) (19). CINeMA contains six domains: within-study bias, between-study bias, indirectness, imprecision, heterogeneity, and inconsistency to grade the quality of evidence. Eventually, the quality of evidence is divided into four levels: high, medium, low, and very low quality.

## 3 Results

### 3.1 Results of literature screening

**Figure 1:**
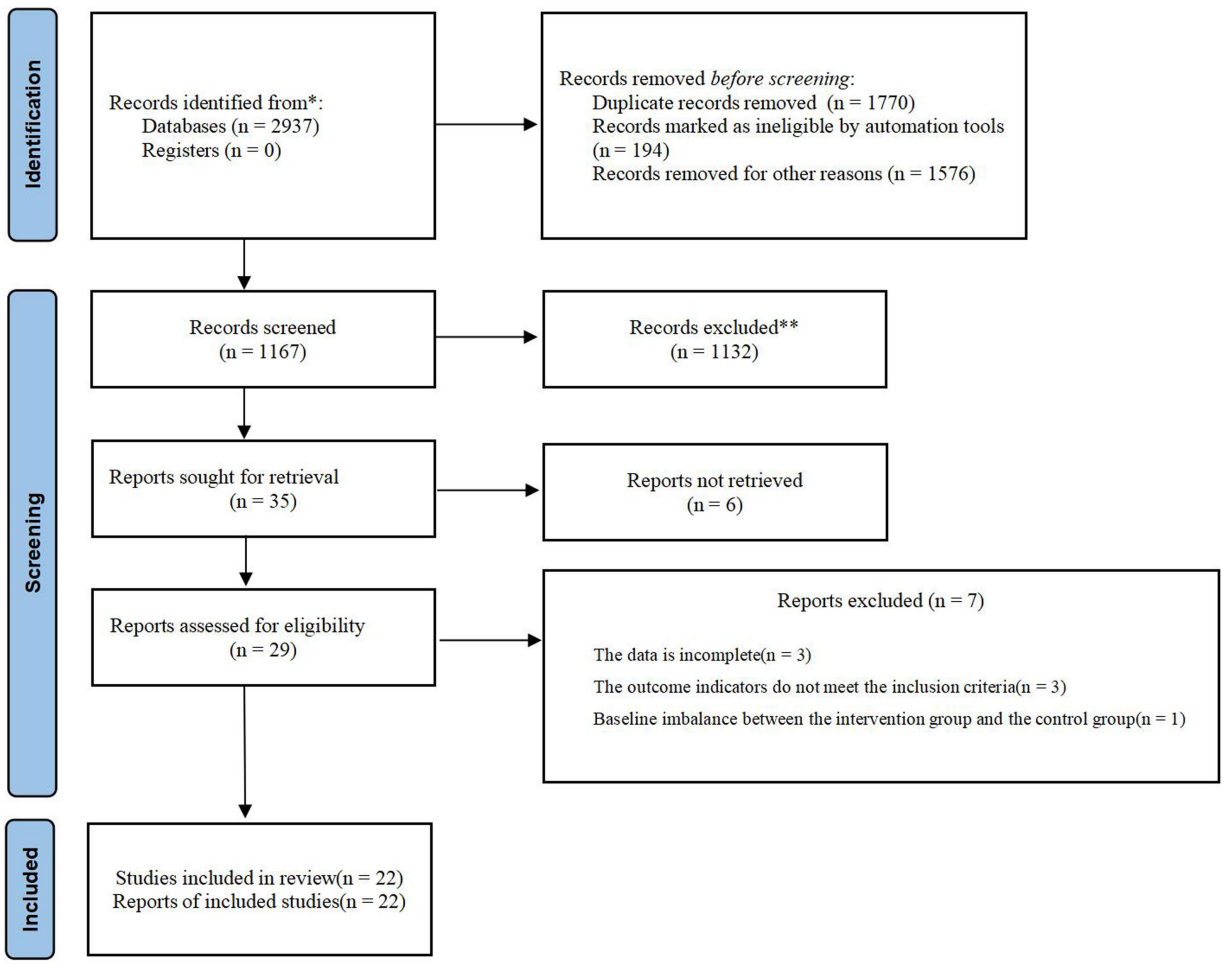
PRISMA flow diagram of the literature screening and selection process.

A total of 2,937 literature were retrieved, 1,770 duplicate literatures were removed, 7 literature were excluded by reading the full text, and 22 literature were finally included (20–41) (Figure 1).

### 3.2 Basic characteristics of included studies

This study included 22 randomized controlled trials (20–41) involving 1,888 patients. Among them, 3 were three-arm trials, and 19 were two-arm trials. The treatment duration in each study ranged from 2 to 8 weeks, and all studies conducted efficacy evaluations after the treatment ended. Since the original studies did not clearly report the time interval from patient discharge to the start of treatment, this information could not be extracted. The included studies involved eight types of acupuncture therapies, including acupuncture (A), fire needle (FN), thumbtack needle (TN), moxibustion therapy (MT), traditional Chinese medicine (TCM), fire needle (FN), functional acupuncture (FA), point application (PA), scalp acupuncture (SA), and their combined regimens. From the perspective of acupoint selection, Hegu (32 times), Dicang (30 times), Jiaqu (28 times), Sishui (22 times), and Xianle (22 times) were the most commonly used acupoints, reflecting the principle of combining local acupoint selection with distant meridian acupoint selection. In terms of the efficacy evaluation criteria, nine studies used the House–Brackmann (HB) grading system to assess the total effective rate (TER), 10 studies used the “Chinese Medicine Disease Syndrome Diagnosis and Therapeutic Effect Standards” (TCM) to assess TER, and 15 studies reported the facial disability index (FDI) (Table 1).

**Table 1:** The basic characteristics included in the study.

Author	Year	Intervention (T/C)		Acupoint (T/C)		Frequency	Sample size (T/C)		Age (T/C)		Course of the disease (T/C)		Time	Outcomes	Criterion of therapeutical effect
Zoukh (20)	2025	EAR+A	A	Shenmen, subcortical, sympathetic, face and cheek	Yangbai, Dicang, Cheek Che, Hegu, Taichong and Fengchi	Once a day	35	35	62.4 ± 8.7	61.8 ± 9.1	3.5 ± 1.2 m	3.7 ± 1.3 m	4w	TER, FDI	TCM
Zhangxx (21)	2025	PNT + MT	A	Dicang, Shuigou, Zygoliao, Cheek Che, Taiyang, Xiaguan	Dicang, Jiche, Zygoliao, Taiyang, Xiaguan, Hegu	Once a day	31	31	62.97 ± 11.19	62.29 ± 12.66	24.42 ± 20.09d	22.87 ± 19.92d	6w	TER, FDI	HB
Zhangll (22)	2023	TN	A	Shuigou, Neiguan, Sanyinjiao, Jiquan, Chize, Weizhong, Hegu	Shuigou, Neiguan, Sanyinjiao, Jiquan, Chize, Weizhong	Ten times a day	39	38	62.77 ± 7.12	62.18 ± 8.43	3.25 ± 0.56d	2.05 ± 0.32d	2w	TER, FDI	HB
Zhangjy (23)	2024	A + CT	CT	Fengchi, Yifeng, Baihui, Hegu, Dicang, Yingxiang, Xiaguan, Sibai, Zygoliao, Quchi		Once every 2 days	30	30	61.48 ± 5.14	62.43 ± 4.67			4w	TER	HB
Yuyy (24)	2023	C + A	A	Zygoliao, Yingxiang, Dicang, Cheek car, Chengzhi, Xiaguan, Shuigou, Hegu, Zusanli		Once a day	30	30	62.2 ± 5.6	64.2 ± 6.6	9.5 ± 4.9w	8.7 ± 5.4w	3w	TER	TCM
Xuzj (25)	2018	A + TCM/A/TCM		Yangbai, Sibai, Zygoliao, Jiche, Dicang, Hegu, Zusanli, Neiguan, Sanyinjiao, Shuigou		Once a day	40/40/40		45.16 ± 6.76/46.56 ± 7.45/47.12 ± 7.48		21.03 ± 5.23/22.24 ± 6.36/20.66 ± 5.02d		4w	TER, FDI	HB
Xumz (26)	2020	A + FN	FN	Sibai, Chengqi, Zygoliao, Dazhui, Taiyang, Yingxiang, Qiche, Zusanli, Hegu, Zhongwan, Dicang, Jexi, Tianshu, Shenting, Sizhukong, Baihui	Fengchi, Yifenghe Baihui, Dicang, Xiaguan, Sibai, Zygoliao, Hegu, Quchi	Once every 2 days	50	50	63.11 ± 6.32	63.23 ± 6.40	37.40 ± 5.39d	37.53 ± 5.47d	2w	FDI	HB
Xiesp (27)	2017	FN	A	Baihui, Yifeng, Fengchi, Xiaguan, Dicang, Zygoliao, Sibai, Quchi, Hegu	Neiguan, Shuigou, Sanyinjiao, Jiquan, Weizhong, Chize	Once every 2 days	54	54	62.74 ± 10.98	62.79 ± 11.05	39.62 ± 14.39d	39.50 ± 14.16d	3w	TER, FDI	TCM
Wangyz (28)	2022	A + TCM	A	Dazhui, Shiting, Baihui, Zhongwan, Zusanli, Jexi, Tianshu, Sibai, Dicang, Zygoliao, Yingxiang, Chengqi, Hegu		Once a day	49	49	58.4 ± 10.5	57.6 ± 10.2	23.7 ± 4.8d	24.4 ± 5.0d	4w	FDI	
Wangn (29)	2020	A + TCM	CT	Yangbai, Sibai, Qiche, Hegu, Neiguan, Zygoliao, Shuigou, Zusanliyi, Sanyinjiao		Once a day	47	47	48.7 ± 3.2	48.4 ± 3.6	19.7 ± 1.5d	19.4 ± 1.6d	4w	TER, FDI	HB
Tanj (30)	2022	A + CT	CT	Hegu, Taichong, Dicang, Qiche		Once a day	40	40	55.47 ± 7.91	56.28 ± 7.89	1.27 ± 0.38 m	1.29 ± 0.35 m	4w	TER, FDI	HB
Suny (31)	2025	FA	A	Yangbai, Juliao, Jiche, Xiaguan, Qianzheng, Dicang, Yifeng, Hegu, Baihui		Once a day	40	40	65.5 ± 6.25	64.4 ± 5.85	14.25 ± 3.37d	14.45 ± 3.15d	6w	TER	TCM
Pengk (32)	2024	A + FN	FN	Sibai, Chengqi, Zygoliao, Dazhui, Taiyang, Yingxiang, Qiche, Zusanli, Hegu, Zhongwan, Dicang, Jexi, Tianshu, Shenting, Sizhukong, Baihui	Fengchi, Yifeng, Baihui, Dicang, Xiaguan, Sibai, Zygoliao, Hegu, Quchi	Once every 2 days	60	60	63.44 ± 6.26	62.58 ± 6.32	44.89 ± 5.11d	45.45 ± 5.32d	8w	FDI	
Lucj (33)	2024	TN + CT	CT	Yingxiang, Sibai, Dicang, Cheek car, Zygoliao, Xiaguan, Yifeng	Dicang, Qiche, Zygoliao, Yingxiang, Sibai, Fengchi, Shenting, Baihui, Hegu	Once a day	30	30	62.13 ± 8.90	60.79 ± 8.52	35.53 ± 24.89d	33.23 ± 20.30d	4w	TER, FDI	HB
Liuys (34)	2020	A + TCM	A	Sishencong, Shuigou, Sibai, Yingxiang, Dicang, Cheek car, Yifeng, Hegu, Zusanli, Neiguan, Sanyinjiao		Once a day	54	53	55.4 ± 6.2	54.1 ± 6.6	27.2 ± 4.9d	26.7 ± 4.9d	4w	TER, FDI	TCM
Liubl (35)	2024	C + A	A	Yifeng, Xiaguan, Chengzhi, Dicang, Jiche, Yangbai, Sibai		Once a day	60	60	78.30 ± 6.40	77.22 ± 6.44	3.02 ± 0.51 m	3.00 ± 0.55 m	6w	TER, FDI	TCM
Liaoyb (36)	2024	A + Rood/A/Rood		Sanli, Hegu, Taichong, Baihui, Shuigou, Sibai, Xiaguan, Dicang, Cheek car, Wind		Once a day	30/30/30		61.00 ± 12.89/60.06 ± 11.55/62.13 ± 9.95		20.00 ± 6.23/21.63 ± 5.17/21.13 ± 5.77d		3w	TER, FDI	HB
Liangxm (37)	2019	A + TCM	CT	Baihui, Hegu, Zusanli, Dicang, Qiche, Zygoliao, Xiaguan, Philtrum		Once a day	49	49	53.26 ± 4.57	4.27 ± 5.11	21.25 ± 3.92d	20.54 ± 3.64d	4w	TER	FDI
Liye (38)	2020	A + MT	A	Yifeng, Yingxiang, Yangbai, Qiche, Dicang, Hegu		Once a day	52	52	55.65 ± 6.77	55.87 ± 6.95	2.24 ± 0.36 m	2.34 ± 0.40 m	8w	TER	TCM
Zengxb (39)	2017	PA + CT	CT			Once a day	30	30	54.6 ± 13.5	59.2 ± 10.7	21 ± 2.9d	18 ± 3.5d	4w	TER	TCM
Zengj (40)	2017	BTTA/A/BTTA+A		Hegu, Cheek car, Zygoliao, Xiaguan, Zusanli		Twice a day	40/40/40		55.07 ± 5.98/55.18 ± 6.17/54.63 ± 6.79/54.31 ± 6.37					TER, FDI	TCM
Dingjh (41)	2017	SA	A	Qianzheng, Yangbai, Cuanzhu, Yingxiang, Dicang, Qiche, Quchi, Hegu, Waiguan, Zusanli, Xuehai, Fenglong		Once a day	29	28	56.90 ± 10.44	57.54 ± 8.43	5.38 ± 3.68d	6.18 ± 4.73d	4w	TER	TCM

T, treatment group; C, control group; PNT, Penetrating needling technique; MT, moxibustion therapy; TN, thumbtack needle; CT, conventional therapy; TCM, traditional Chinese medicine; FN, fire needle; FA, Functional acupuncture; C, cupping; PA, point application; BTTA, Botulinum toxin type A; SA, scalp acupuncture; HB, House Brackmann.

### 3.3 Assessment of publication bias

**Figure 2:**
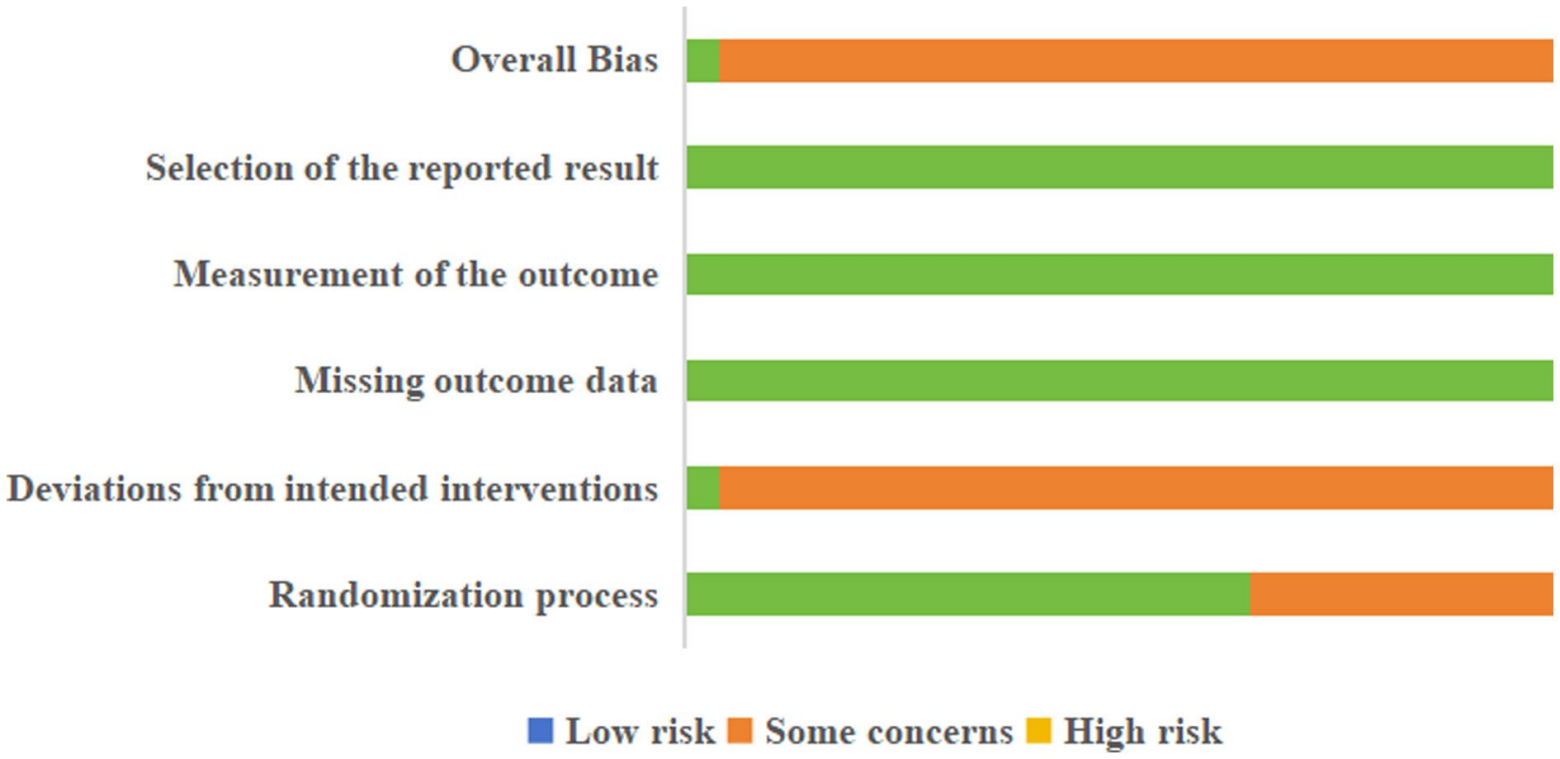
Risk of bias assessment summary for the included randomized controlled trials (RoB2).

A total of 14 studies described the correct method of random-sequence generation; Blinding was difficult to implement due to the specific nature of acupuncture. One study (37) mentioned blinding of subjects and assessors, whereas the remaining literature did not mention the blinding of subjects. The allocation concealment of all studies did not mention the specific implementation scheme. There was no selective reporting or missing results in the included studies. No other risks of bias were described in detail. One study was assessed as low risk (37), and the rest were assessed as unknown risk (Figure 2).

### 3.4 Meta-analysis

#### 3.4.1 TER

##### 3.4.1.1 HB

**Figure 3:**
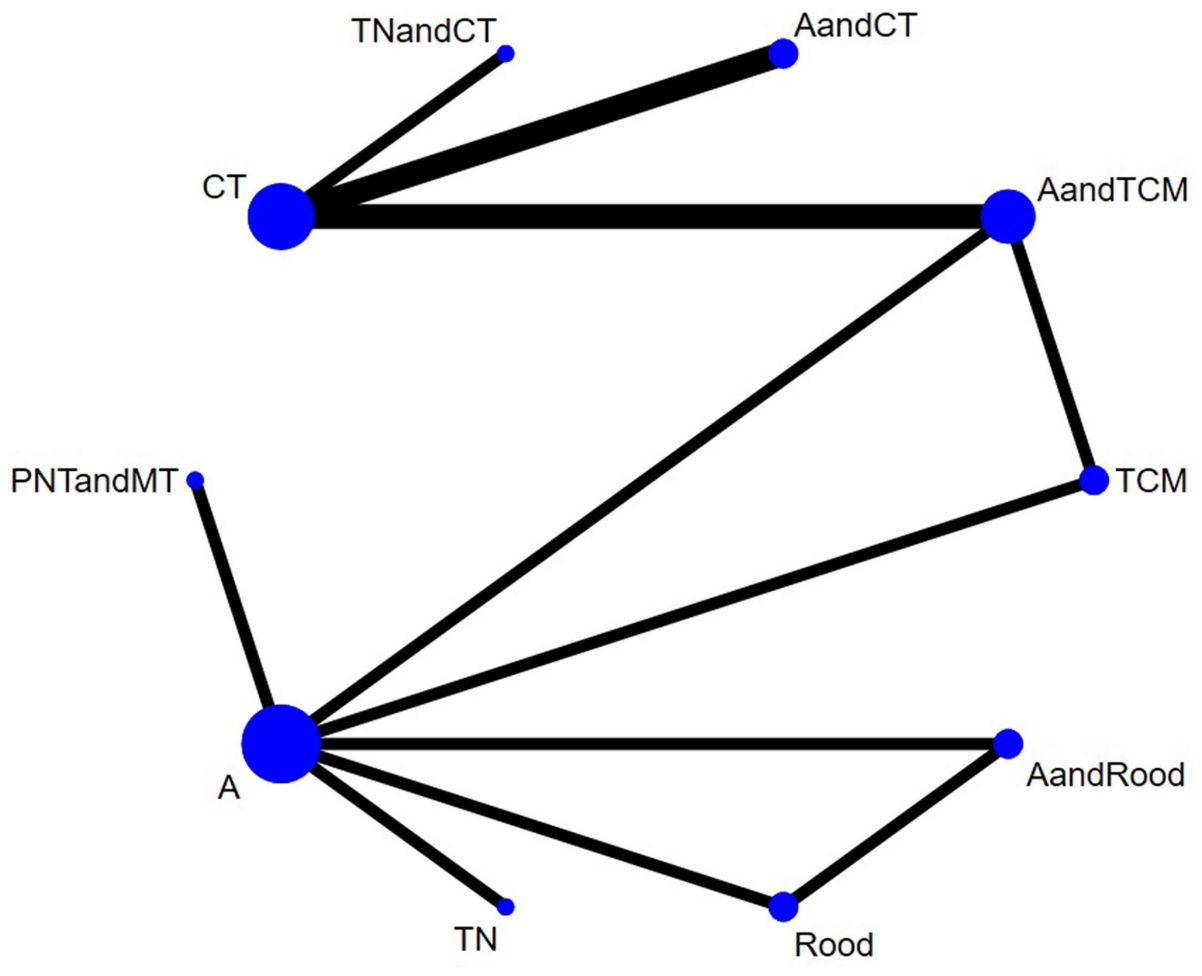
Network plot of the included interventions for the outcome of TER (HB).

**Figure 4:**
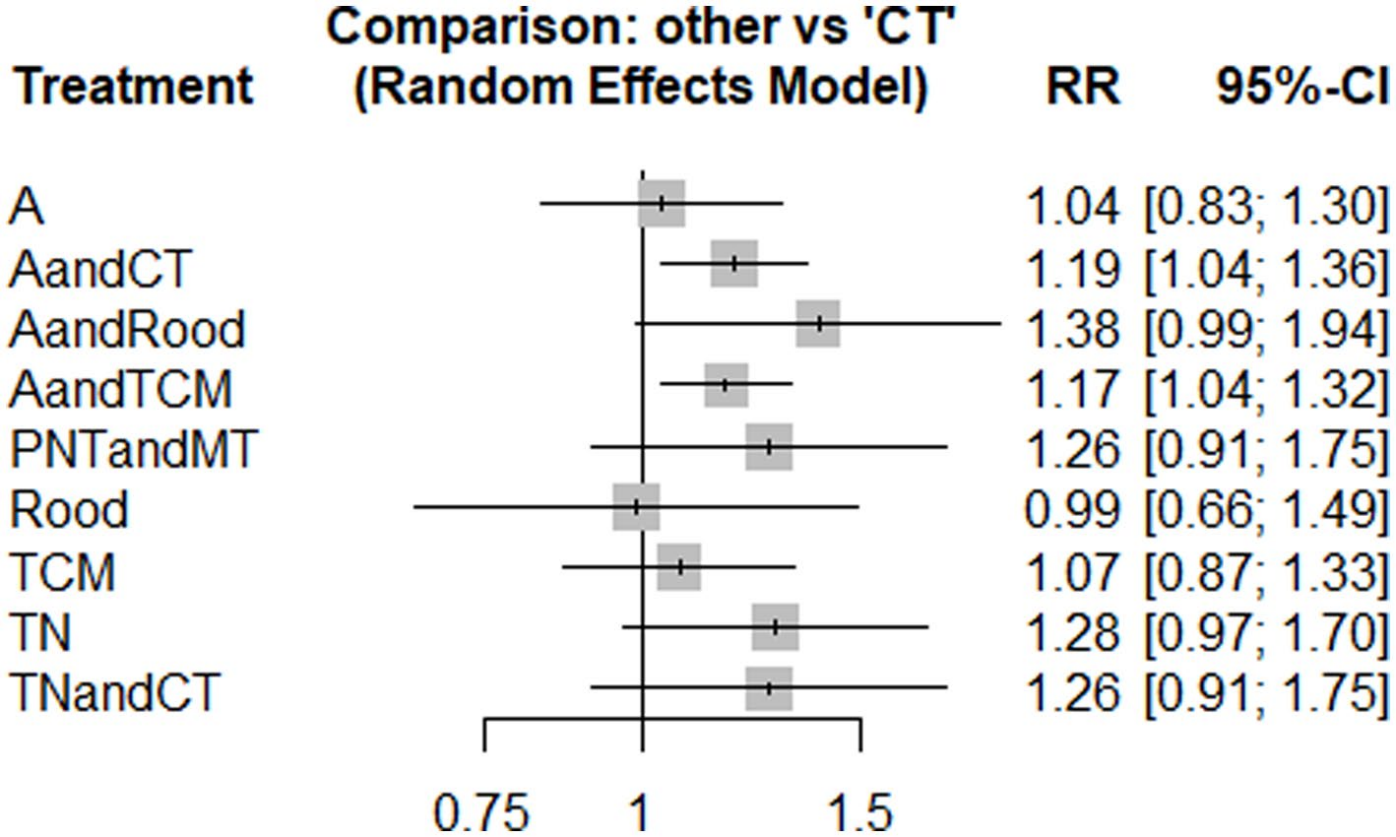
Forest plot of pairwise comparisons for the outcome of TER (HB).

Nine studies used HB. The network diagram contained closed loops. Using the inconsistency model for analysis, the results showed no statistical significance (*p* > 0.05) and no obvious inconsistency. Therefore, the consistency model was used (Figure 3). The heterogeneity test results showed that the heterogeneity among the studies was small (*I*^2^ = 7%). The model’s convergence diagnostic was good. The results of the network meta-analysis showed that compared with CT, the effects of AandCT and AandTCM had significant advantages (*p* < 0.05) (Figure 4; Supplementary material – 2. League table). The top 3 ranked by SUCRA were: AandCT (0.84), AandTCM (0.73), and PNTandMT (0.68) (Table 2).

**Table 2:** SURCA sorting result.

Intervening measure	TER				PDI	
	HB		TCM			
	SUCAR	No	SUCAR	No	SUCAR	No
A	0.23	8	0.24	10	0.21	15
AandCT	0.84	1			0.34	12
AandMT			0.48	8		
AandRood	0.58	5			0.38	10
AandTCM	0.73	2	0.79	2	0.56	7
BTTA			0.25	9	0.51	8
BTTAandA			0.68	5	0.90	1
CandA			0.52	6	0.81	2
CT	0.15	10	0.01	12	0.03	16
EAandCT						
EARandA			0.68	4	0.31	13
FA			0.51	7		
FN			0.82	1	0.38	9
PAandCT			0.17	11		
PNTandMT	0.68	3			0.79	3
Rood	0.22	9			0.36	11
SA			0.78	3		
TCM	0.32	7			0.24	14
TN	0.55	6			0.65	6
TNandCT	0.66	4			0.79	4
AandFN					0.66	5

PNT, Penetrating needling technique; MT, moxibustion therapy; TN, thumbtack needle; CT, conventional therapy; TCM, traditional Chinese medicine; FN, fire needle; FA, Functional acupuncture; C, cupping; PA, point application; BTTA, Botulinum toxin type A; SA, scalp acupuncture.

##### 3.4.1.2 TCM

**Figure 5:**
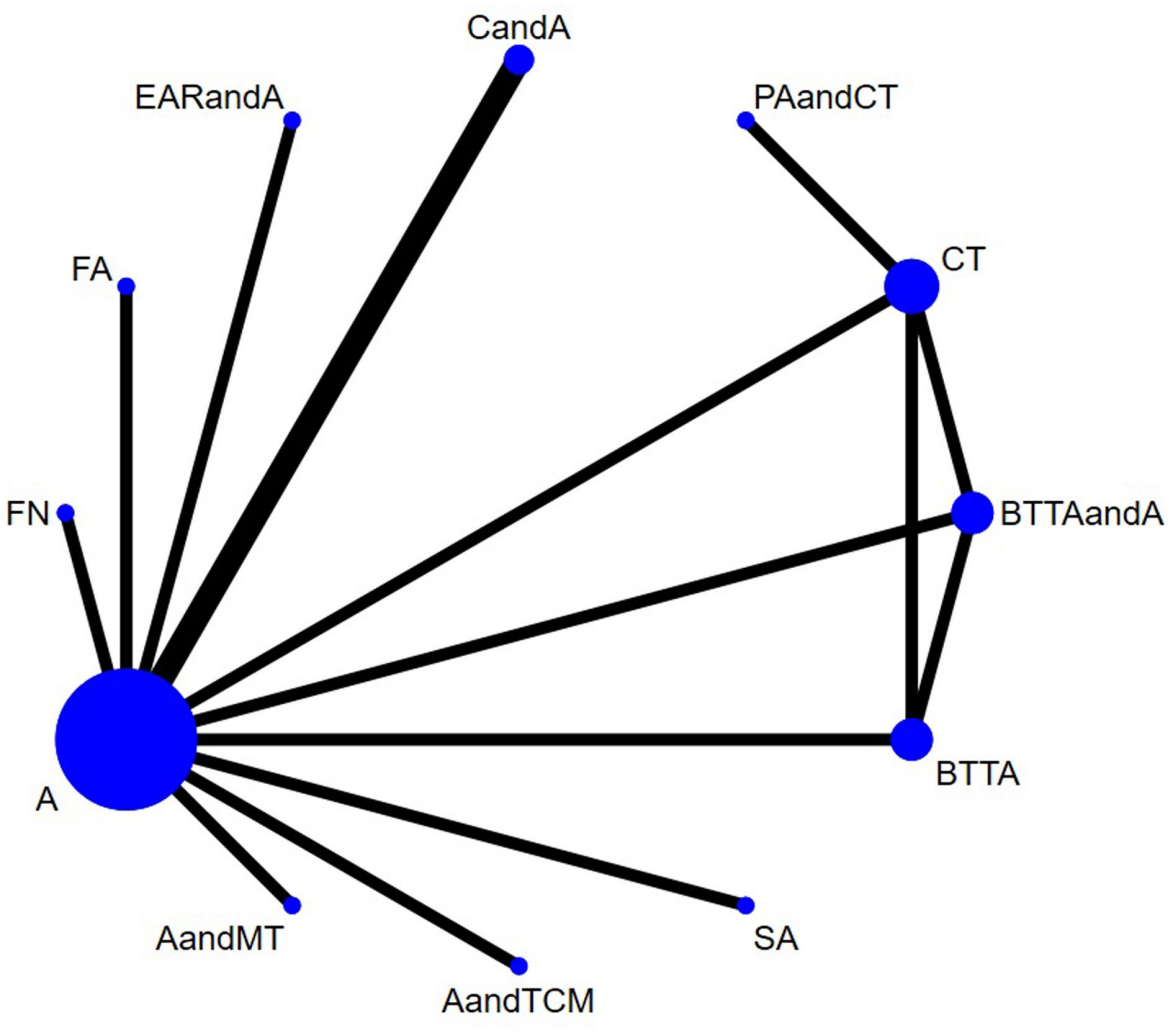
Network plot of the included interventions for the outcome of TER (TCM).

**Figure 6:**
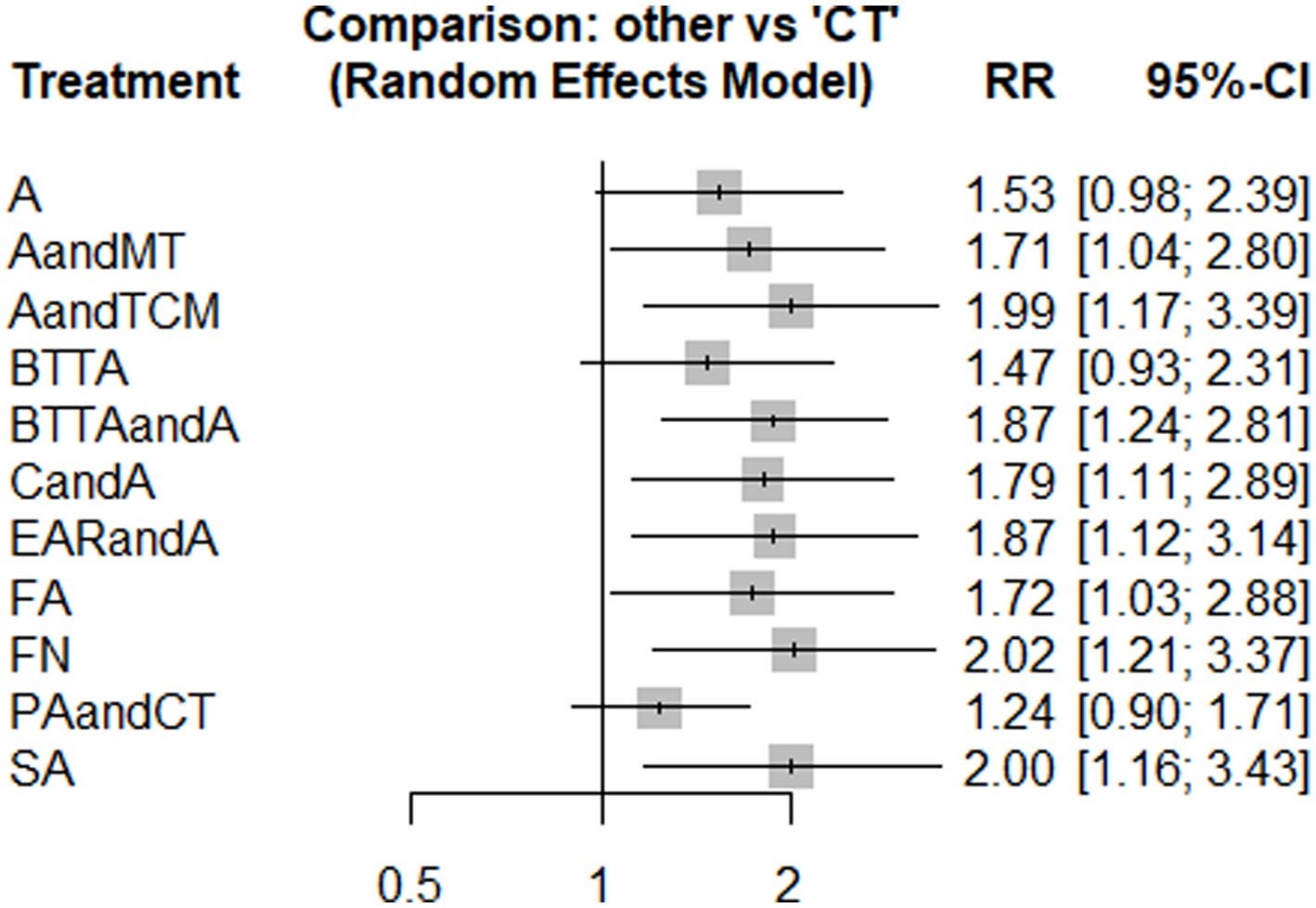
Forest plot of pairwise comparisons for the outcome of TER (TCM).

Ten studies utilized TCM. The network diagram contained closed loops. Using the inconsistency model for analysis, the results showed no statistical significance (*p* > 0.05) and no obvious inconsistency. Therefore, the consistency model was used (Figure 5). The heterogeneity test results indicated that the heterogeneity among the studies was small (*I*^2^ = 5%). The model’s convergence diagnostic was good. The results of the network meta-analysis showed that compared with CT, AandMTT, AandTCM, BTTAandA, CandA, EARandA, FA, FN, and SA had significant advantages (*p* < 0.05) (Figure 6; Supplementary material – 2. League table). The top 3 ranked by SUCRA were FN (0.82), AandTCM (0.79), and SA (0.78) (Table 2).

#### 3.4.2 PDI

**Figure 7:**
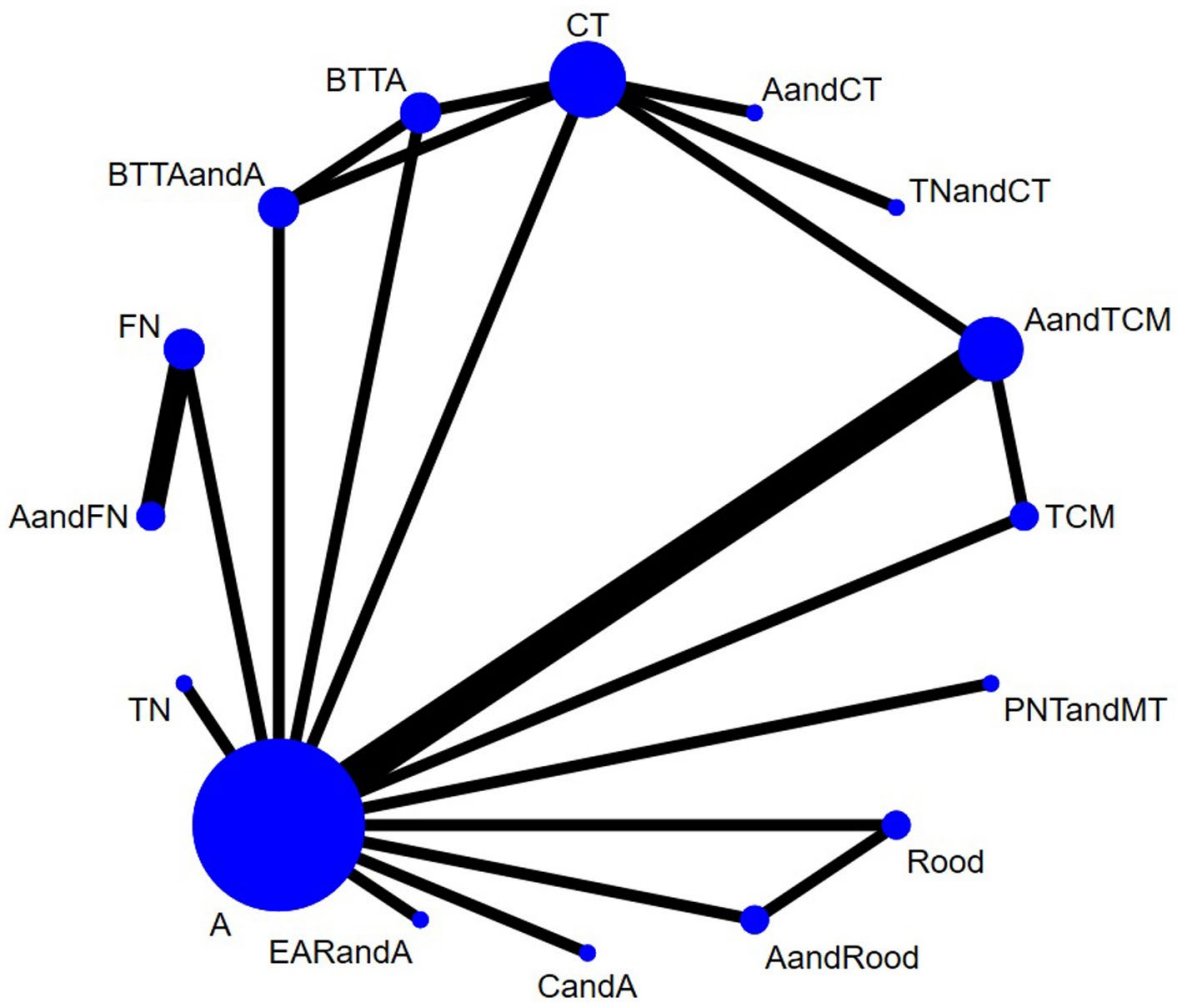
Network plot of the included interventions for the outcome of FDI.

**Figure 8:**
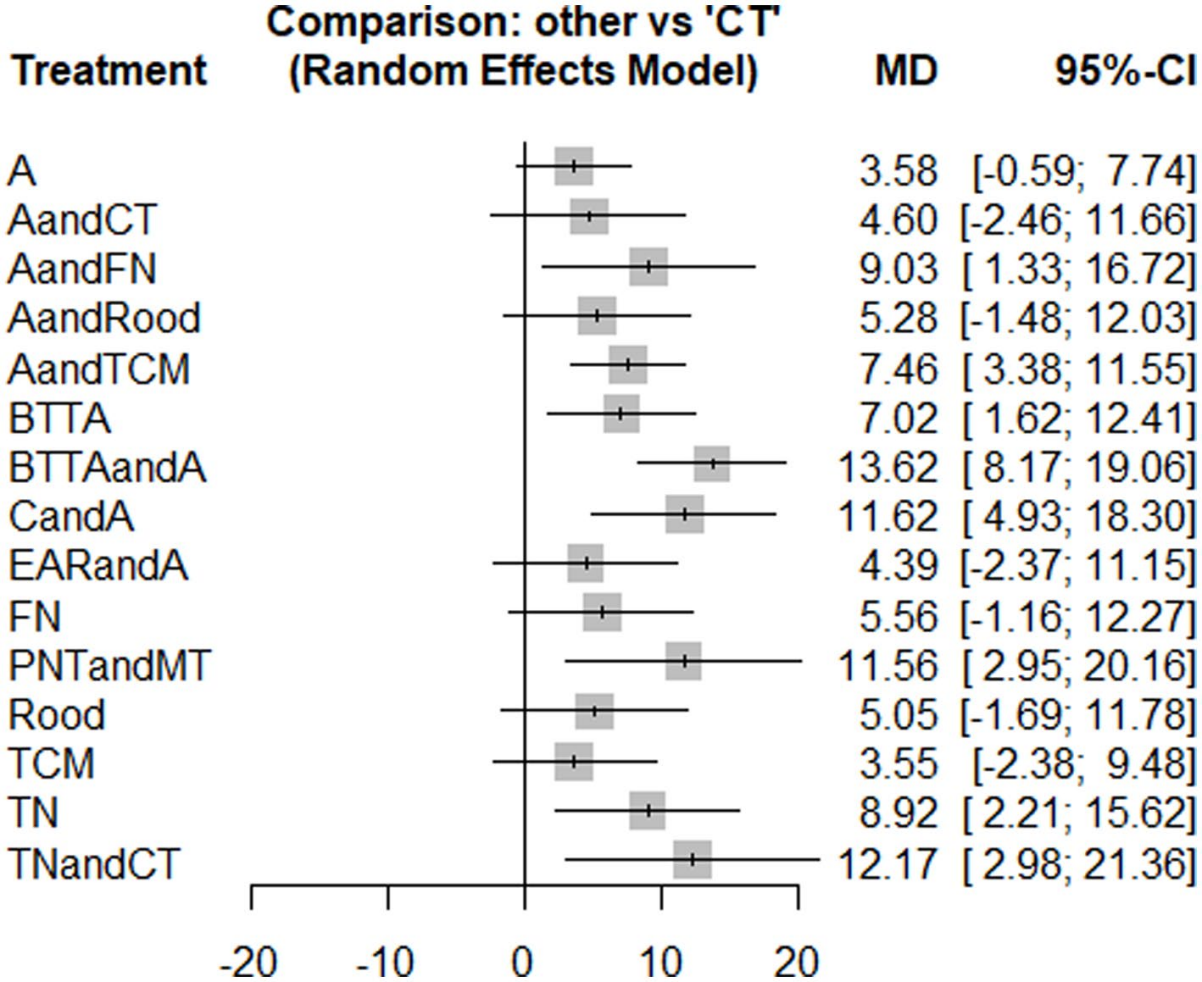
Forest plot of pairwise comparisons for the outcome of FDI.

PDI was reported in 15 studies. There were closed loops in the reticular plots, which were analyzed using the inconsistency model, and the results showed no statistical significance (*p* > 0.05). No obvious inconsistency was found, so the consistency model was used (Figure 7). The results of the heterogeneity test showed that the heterogeneity between studies was small (*I*^2^ = 3%). The model’s convergence diagnostic was good. Network meta-analysis results showed that compared with CT, AandFN, AandTCM, BTTA, AandBTTA, CandA, PNTandMT, TN, the effect of TNandCT showed a significant advantage (*p* < 0.05) (Figure 8; Supplementary material – 2. League table). The top 3 SUCRA results were BTTAandA (0.90), CandA (0.81), and PNTandMT (0.79) (Table 2).

### 3.5 Results of publication bias analysis

**Figure 9:**
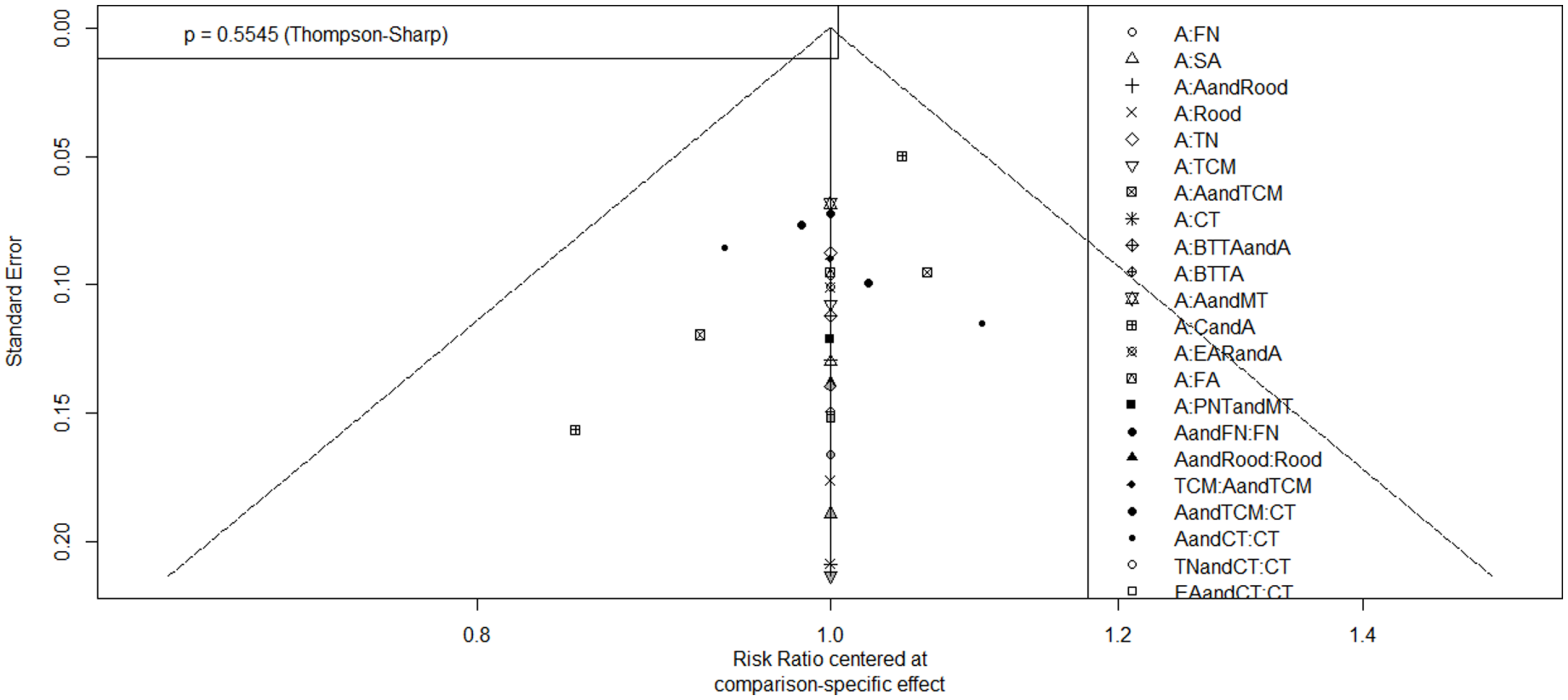
Funnel plot for the assessment of publication bias (TER).

**Figure 10:**
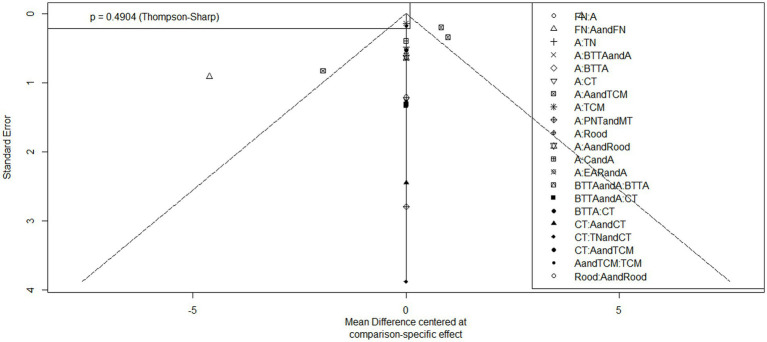
Funnel plot for the assessment of publication bias (FDI).

The results of the meta-analysis were tested for publication bias, and the results showed that there was little possibility of publication bias (*p* > 0.05) (Figures 9, 10).

### 3.6 Meta-regression

Meta-regression was performed on frequency and treatment duration to discuss the influence of these factors on the efficacy. The results showed no significant correlation between treatment frequency and duration, and efficacy compared with the control group; and the results were not statistically significant (Table 3).

**Table 3:** Meta-regression.

Outcome indicator	Intervening measure	Frequency [RR/MD (95%CI)]	Course [RR/MD (95%CI)]
TER	A.AandMT	0.99 (−1.89, 4.18)	0.54 (−13.87, 21.34)
	A.AandRood	1.81 (−1.24, 6.77)	2.09 (−4.89, 9.16)
	A.AandTCM	0.66 (−1.17, 3.15)	0.75 (−0.71, 2.17)
	A.BTTA	0.22 (−3.98, 5.54)	0.09 (−3.02, 2.42)
	A.BTTAandA	2.08 (−1.51, 9.65)	1.93 (−0.39, 9.17)
	A.CandA	1.50 (−3.66, 4.59)	1.75 (0.26, 3.49)
	A.CT	−0.70 (−1.94, 0.52)	−0.72 (−2.04, 0.57)
	A.EARandA	1.60 (−2.16, 4.81)	1.77 (−0.79, 4.65)
	A.FA	0.68 (−2.98, 3.78)	0.54 (−14.42, 14.17)
	A.FN	1.30 (−8.06, 5.65)	1.80 (−7.40, 7.04)
	A.PNTandMT	1.03 (−22.48, 4.36)	0.93 (−12.10, 10.34)
	A.Rood	−0.29 (−2.98, 2.52)	−0.20 (−12.81, 5.08)
	A.SA	1.62 (−2.22, 4.90)	1.87 (−0.60, 4.76)
	A.TCM	0.00 (−3.03, 5.71)	0.19 (−1.92, 2.66)
	A.TN	6.71 (−19.23, 37.15)	3.05 (−7.04, 14.11)
	CT.AandCT	1.40 (−1.38, 3.49)	1.63 (−0.45, 3.60)
	CT.EAandCT	1.79 (−0.83, 5.02)	1.72 (−33.65, 11.36)
	CT.PAandCT	1.03 (−7.41, 5.12)	1.11 (−1.03, 3.60)
	CT.TNandCT	0.85 (−4.52, 3.62)	0.88 (−1.51, 3.36)
	FN.AandFN	1.99 (−3.57, 16.24)	2.42 (−13.14, 23.73)
PDI	A.AandRood	−1.05 (−23.48, 20.63)	−1.31 (−38.33, 37.44)
	A.AandTCM	0.58 (−30.26, 11.05)	3.48 (−10.16, 18.10)
	A.BTTA	4.43 (−12.82, 26.96)	2.78 (−24.56, 27.23)
	A.BTTAandA	10.92 (−7.60, 28.78)	9.64 (−14.71, 34.47)
	A.CandA	5.79 (−10.78, 22.30)	20.22 (−26.02, 82.57)
	A.CT	−3.79 (−13.37, 5.70)	−4.21 (−23.12, 14.73)
	A.EARandA	−2.05 (−20.44, 14.97)	0.77 (−24.68, 33.24)
	A.FN	−1.31 (−36.91, 21.06)	4.15 (−22.35, 36.54)
	A.PNTandMT	5.21 (−30.15, 26.74)	13.72 (−42.73, 96.03)
	A.Rood	−1.17 (−19.59, 20.21)	−1.57 (−34.57, 29.69)
	A.TCM	−2.76 (−30.66, 12.33)	−2.23 (−31.35, 21.95)
	A.TN	58.33 (−224.92, 236.61)	−9.84 (−141.40, 41.40)
	CT.AandCT	4.58 (−16.48, 24.50)	4.53 (−22.54, 30.84)
	CT.TNandCT	12.24 (−8.81, 37.95)	12.03 (−15.92, 38.82)

### 3.7 Results of quality of evidence evaluation

A total of 13 pieces of evidence were evaluated, including 1 piece of high-quality evidence, 8 pieces of medium-quality evidence, and 4 pieces of low-quality evidence. Overall high- and medium-quality evidence accounted for 70%. The main reasons for downgrading were incoherence and heterogeneity (Supplementary material – 3. Results of the quality assessment of evidence).

## 4 Discussion

This study conducted a network meta-analysis to systematically evaluate the clinical efficacy of 8 acupuncture therapies (including combined regimens) in treating central facial paralysis after stroke. A total of 22 randomized controlled trials were included, involving 1,888 patients. Regarding the total effective rate (TER), because the original studies used different efficacy assessment criteria, we conducted subgroup analyses for studies using the HB standard and the TCM standard separately. The 9 studies using the HB standard showed that compared with conventional treatment (CT), acupuncture combined with conventional treatment (AandCT, SUCRA = 0.84) and acupuncture combined with traditional Chinese medicine (AandTCM, SUCRA = 0.73) had significant therapeutic advantages; the 10 studies using the TCM standard showed that fire needle (FN, SUCRA = 0.82), acupuncture combined with traditional Chinese medicine (AandTCM, SUCRA = 0.79), and scalp acupuncture (SA, SUCRA = 0.78) ranked at the top. In terms of the improvement of the facial disability index (FDI), 15 studies showed that botulinum toxin type A combined with acupuncture (BTTAandA, SUCRA = 0.90), cupping combined with acupuncture (CandA, SUCRA = 0.81), and penetrating needling technique combined with moxibustion (PNTandMT, SUCRA = 0.79) had the best therapeutic effects. Overall, BTTAandA had a prominent advantage in improving patients’ subjective function, while AandTCM, FN, and SA performed well in improving the objective efficacy. From the perspective of acupoint selection, the included studies generally adopted core acupoints such as Dicang penetrating Cheijue, Hegu, Yangbai, Sishui, Yiweng, and Xiaokuan, reflecting the principle of combining local acupoint selection with distant meridian acupoint selection. The treatment course was 4 weeks long and could be extended to 6–8 weeks depending on the condition. Meta-regression analysis did not show a significant correlation among treatment frequency, course of treatment, or efficacy, but most high-ranking intervention measures had treatment courses of more than 4 weeks, suggesting that an adequate treatment cycle may be a key to achieving good efficacy. In terms of safety, the adverse reactions reported in the included studies were mainly local pain, ecchymosis, dizziness, nausea, etc., which were mostly transient, and rare severe complications were observed. This indicates that acupuncture and its combined therapies are safe. The Eeidence quality evaluation showed that approximately 70% of the evidence was of moderate or higher quality, and the results were reliable. The publication bias test showed no significant bias, and the heterogeneity and inconsistency test results were not statistically significant, enhancing the stability of the conclusion.

The mechanism of acupuncture in the treatment of facial paralysis may be to stimulate facial nerve repair, improve blood circulation, regulate immune imbalance, enhance muscle contraction, and accelerate facial paralysis recovery through central regulation (42). Acupuncture can activate the trigeminal neural-facial reflex pathway by stimulating specific acupoints and promote axonal regeneration of damaged nerves (9). In terms of acupoint selection, Decang and buccal che are important acupoints for the treatment of facial paralysis, and penetrating needling of these two acupoints can enhance therapeutic effect. Hegu (LI4) is a special acupoint for treating facial and oral diseases. Zusanli (ST 36) is a commonly used acupoint for strong health care in clinical practice, which can treat facial paralysis by regulating the circulation of qi and blood on the head and face. It also works with Sibai, Xiaguan, Quchi, and other acupoints to play a role together. Clinical evidence showed that acupuncture therapy could improve the House–Brackmann (HB) facial nerve grading system score [MD = 6.41, 95% CI (3.69, 9.14), *Z* = 4.62, *p* < 0.05, *p* < 0.00001], FDI score of body function [MD = 2.72, 95% CI (0.31, 5.12), *Z* = 2.21, *p* = 0.03], total clinical effective rate [RR = 1.25, 95% CI (1.15, 1.35), *p* < 0.000 01, *Z* = 5.57, *p* < 0.00001] was higher than conventional drug and exercise rehabilitation therapy, suggesting that acupuncture therapy has certain advantages in the treatment of CFP after stroke (9). As a special form of acupuncture and moxibustion, the thermal effect of fire needling therapy can enhance local microcirculation and accelerate the functional reconstruction of the neuromuscular junction by up-regulating the expression of vascular endothelial growth factor (VEGF) and nerve growth factor (NGF) (43). According to traditional Chinese medicine theory, these two therapies regulate the circulation of qi and blood and improve the pathological state of “wind-phlegm blocking collaterals” through the “activating meridians and collaterals” effect (44). At the same time, fire needling also functions to tonify and supplement. Acupuncture at the Yangming and Shaoyang meridians can replenish qi and generate blood, so that blood is sufficient, the facial muscles are relaxed, and the blood volume in brain lesions is improved, which is helpful for the repair of injured nerves and blood vessels. The rapid insertion and withdrawal of fire needling during the treatment process can relieve patients’ pain, reduce tension during needling, and cause little damage to muscles and tissues. Gao’s study (45) found that fire needling for the treatment of refractory facial paralysis can repair damaged nerves, blood vessels, and tissues after two courses, with a total effective rate of 87.5%.

Botulinum toxin type A is a bacterial exotoxin produced by *Clostridium botulinum*. The core mechanism of its therapeutic effect lies in its selective inhibition of acetylcholine release at nerve endings (46). When BTTA is locally injected into muscle tissue, it specifically binds to receptors on nerve endings. It enters cells, cleaving SNARE protein complexes such as SNAP-25, thereby blocking the fusion process between synaptic vesicles and the presynaptic membrane and inhibiting calcium-mediated acetylcholine release (47). This effect produces chemical denervation, causing temporary paralysis of the injected muscle (47). The action of BTTA is highly selective, mainly targeting peripheral cholinergic nerve endings, and it has no direct toxicity to the central nervous system. Its clinical effect usually peaks at 2 weeks after injection, lasts for approximately 3–6 months, and then gradually recovers as nerve endings sprout and synaptic reconstruction occurs (48). From the perspective of neural function, the essence of central facial paralysis after stroke is the functional decline of upper motor neurons, often accompanied by muscle strength imbalance on the healthy side and the affected side, manifested as a shallower nasolabial fold and drooping of the mouth corner in static positions, and bilateral asymmetry worsening in dynamic positions. Traditional rehabilitation training mainly targets muscles on the affected side, but often fails to correct muscle strength imbalance between the sides. The application strategy of BTTA is based on this pathological characteristic: by selectively injecting an appropriate amount of BTTA into the facial muscles on the healthy side, moderately weakening the contraction strength of the healthy side muscles, thereby reducing the functional contrast between the paralyzed muscles on the affected side and the healthy side, and increasing the symmetry of both sides of the face (49). Research by Yang Yuanbin et al. confirmed that adding healthy side BTTA injection to conventional facial training significantly reduced bilateral differences in the distances from the mouth corner to the midline and from the mouth corner to the outer canthus in the resting and active states of the patients after 4 weeks of treatment, suggesting a significant improvement in facial symmetry-3. The advantage of this intervention strategy lies in that it does not interfere with the active rehabilitation training of the affected side muscles and can optimize overall facial function performance by adjusting the strength of the healthy side muscles. Relevant studies also show that appropriately weakening the strength of the healthy side’s facial muscles can promote active movement of the affected side’s facial muscles, thereby promoting recovery of function on the affected side (50). From the rationality of acupoint selection, the included studies (acupoints of BTTA combined with acupuncture mainly include Hegu, Jiachu, Xianliang, Xiaokuan, and Zusanli, etc.), and the selection of these acupoints is based on both traditional Chinese medicine theory and modern anatomy. Hegu is the original acupoint of the Hand Yangming Large Intestine Meridian, which is an important acupoint for treating facial and oral disorders, regulating the circulation of qi and blood in the head and face; Jiachu, Xianliang, and Xiaokuan are local acupoints on the face, directly acting on the facial expression muscle groups; Zusanli is a strengthening and health-preserving acupoint, which can regulate the qi and blood circulation throughout the body. Acupuncture at these acupoints can exert effects such as unblocking meridians, harmonizing qi and blood, and nourishing tendons and muscles (50). For the injection site of BTTA, based on clinical experience in esthetic medicine and neurology, the healthy side injection is usually targeted at muscles involved in smile expression, such as the zygomatic major and zygomatic minor muscles, to improve dynamic symmetry (51). Borodic’s research showed that injecting 10–15 U of BTTA into the zygomatic muscle area can effectively improve facial asymmetry in patients after treatment for facial muscle spasm-8. Modern research emphasizes that BTTA injection requires precise techniques, appropriate doses, and accurate site selection to balance both neurological efficacy and facial esthetic effects (51).

From the perspective of the acupuncture point combinations included in the study, commonly used points include Dicang, Jiaqu, Guanyu, Yangbai, Sibai, Yiwen, Xiaokuan, Yingxiang, etc. These points mostly belong to the Yangming and Shaoyang meridians and have the functions of unblocking facial meridians, regulating qi and blood, and nourishing tendons and muscles. For example, the combination of Dicang and Jiaqu is a classic set of points for treating facial paralysis, which can enhance the transmission of needle sensation and stimulate the meridian qi; Guanyu is a key point for “face and mouth Guanyu collection” and can regulate the qi and blood in the head and face; local points such as Yangbai and Sibai can directly stimulate the affected muscle groups. At the technical level, fire needle therapy generates a thermal effect through high-temperature incision, which can dilate local blood vessels, improve microcirculation, promote the expression of nerve growth factors, and thereby accelerate nerve repair (52); the treatment frequency is mostly once a day or once every other day, and the treatment course is concentrated at 2–8 weeks. Although the meta-regression analysis did not show a significant correlation between treatment frequency, treatment course, and therapeutic efficacy, the treatment courses of most high-ranking intervention measures are above 4 weeks, suggesting that an adequate treatment cycle may be the basis for achieving good therapeutic effects. Future research should further standardize the combination of acupuncture points, acupuncture techniques, and treatment parameters to optimize the clinical plan.

Compared with conventional treatment, acupuncture has many advantages. First, acupuncture is safer. Most adverse events of acupuncture are local hematoma and pain caused by puncturing the skin, or mild reactions such as nausea and dizziness. Serious complications are extremely rare, and most patients can be relieved by rest (53) (Table 4). At the same time, the economic cost of acupuncture is low. Acupuncture treatment accounts for 48.05% of the total medical cost of facial paralysis patients. Still, it can optimize overall medical costs by shortening the length of hospital stay (standardized coefficient 0.697) (54) and reducing the economic burden. Finally, patients’ acceptance and satisfaction were high. A case report on acupuncture combined with tuina therapy reported by Korean scholars shows that integrated therapy can improve the grade of facial nerve function by one level and significantly improve patient satisfaction (55). At present, acupuncture is increasingly recognized internationally. We searched clinical trial registration platforms and found many acupuncture trials for FP were registered (e.g., ChiCTR2400083418, ChiCTR2300067643, NCT03592797, and NCT06393231). We believe that the development trend of acupuncture and moxibustion therapy is toward the precision of treatment methods (clarifying the targets of acupuncture and moxibustion for central nerve remodeling using functional magnetic resonance imaging [fMRI] and other technologies) and the standardization of research methods (establishing a unified efficacy evaluation index system). The normalization of multidisciplinary collaboration and multidisciplinary interdisciplinarity (such as integration with rehabilitation medicine, sports medicine, and psychology).

**Table 4:** Adverse reactions of included studies.

Study	Intervening measure	Adverse reaction (case)
Xumz (25)	A + FN	Headache (1), nausea (1), drowsy (1), dizzy (1)
	FN	Headache (1), nausea (1), drowsy (1), local swelling (1), dizzy (1)
Pengk (32)	A + FN	Pain (2), ecchymoma (2), dizzy (2), headache (1)
	FN	Pain (1), ecchymoma (1), dizzy (2), headache (1)
Liubl (35)	A + C	Nausea (2), headache (1), local swelling (1), drowsy (1), dizzy (1)
	A	Nausea (1), drowsy (1)
Liye (38)	A + MT	Nausea (2), drowsy (1), dry (2), dizzy (1)
	A	Nausea (1), dry (2), dizzy (1)

MT, moxibustion therapy; FN, fire needle; C, cupping.

Based on the 22 randomized controlled trials included in this study, the following clinical application best practice recommendations are proposed: In terms of intervention measures selection, if the House–Brackmann grading is used as the criterion for evaluating efficacy, acupuncture combined with conventional treatment (AandCT) or acupuncture combined with traditional Chinese medicine (AandTCM) are recommended as the priority options; if the “Chinese Medicine Disease Syndrome Diagnosis and Efficacy Standards” is used as the basis, fire needle (FN, SUCRA) or head acupuncture (SA) are recommended as the priority options; when the primary goal is to improve facial function (FDI), botulinum toxin type A combined with acupuncture (BTTAandA) or cupping combined with acupuncture (CandA) are recommended as the priority options. The selection of acupoints is centered on Hegu (32 times), Jizhong (30 times), Jiaqu (28 times), Sishui (22 times), and Xianliang (22 times), with common combination point schemes including Jizhong through Jiaqu, Hegu combined with Taiyang, etc., following the principle of combining local and distal acupoint selection. The treatment frequency is most commonly once a day; fire needles and pressing needles can be once every other day. The treatment course is 4 weeks, which can be extended to 6–8 weeks according to the patient’s condition. In terms of safety, common adverse reactions include local pain, ecchymosis, dizziness, nausea, etc., which are mostly transient. During the operation, the temperature and depth of fire needles, the negative pressure of cupping, and the injection dose of botulinum toxin should be strictly controlled. It is recommended that, in clinical practice, individualized plans be formulated based on the patient’s disease course, severity, and treatment goals, and that the combined use of acupuncture and conventional treatment, traditional Chinese medicine, or modern rehabilitation techniques be emphasized.

The strengths of this study are as follows: Using network meta-analysis, the clinical efficacy of nine different acupuncture and moxibustion therapies for the treatment of post-stroke central facial paralysis was systematically compared, thereby overcoming the limitations of traditional pairwise comparisons and enhancing the statistical power and reliability of the conclusions. Multi-outcome evaluation, taking into account both objective and subjective efficacy: not only the total effective rate (TER) was included as the objective efficacy index, but also the facial disability index (FDI) was used to evaluate the subjective quality of life and functional status of patients, which provided a more comprehensive basis for clinical efficacy. The heterogeneity and bias control were sufficient: consistency tests, heterogeneity analyses, sensitivity analysis, and publication bias tests were used to ensure the stability of the results. The CINeMA tool was used to grade the quality of evidence, which enhanced the credibility of the conclusions. The clinical guidance value is outstanding: the SUCRA ranking identifies interventions with higher efficacy and provides evidence-based support for selecting clinical treatment plans, especially for patients with moderate-to-severe facial paralysis. The economic, safety, and patient acceptance of acupuncture and moxibustion are emphasized in the discussion, which is in line with the current development trend of medical resource optimization and individualized treatment. However, this study also has some limitations; the main issue is heterogeneity, with differences in research design, intervention measures, and outcome assessment. There was wide variation in acupuncture points, stimulation parameters, and treatment cycles. The assessment tools are not uniform (mixed HB classification, Sunnybrook score, etc.). Patients with conflicting directions or magnitudes of effects had subgroup differences in baseline characteristics (e.g., duration and severity of facial paralysis) and in the timing of efficacy assessment (acute vs. recovery). These are potential sources of heterogeneity and incoherence. It should be noted that in this study, acupuncture and moxibustion were classified together under the category of acupuncture therapy. However, there are fundamental differences in their mechanisms of action: acupuncture generates mechanical stimulation by penetrating the acupoints, activating the neural reflex pathways, while moxibustion produces a thermal effect by burning the mugwort, improving local microcirculation. This difference in mechanisms may introduce clinical heterogeneity. However, the number of relevant literature on moxibustion included in this study was relatively small (only three items, all of which were combined intervention measures of acupuncture and moxibustion), and its impact on the overall analysis conclusion was limited. Future research should further distinguish the independent therapeutic effects of acupuncture and moxibustion and explore the synergistic mechanism of their combined application. In the future, standardized protocols for acupuncture operation (such as the World Health Organization [WHO] standard acupoint positioning), unified outcome assessment tools (such as the eFACE system), selective reporting bias reduction through prospective registration study protocols, random-effects model for residual heterogeneity, and meta-regression analysis to explore potential regulatory factors (53) should be established. Although we have analyzed some factors, the evidence is not enough. Research on mechanisms and multidisciplinary integration should be strengthened, and the central and peripheral mechanisms of acupuncture and moxibustion in the treatment of CFP should be clarified through the integration of neuroimaging, molecular biology, and other techniques. Expand real-world research and health economic evaluation, verify the efficacy in a real clinical environment, and conduct cost-effectiveness analysis, so as to provide a basis for the formulation of medical insurance policies and clinical pathways.

## 5 Conclusion

This study conducted a network meta-analysis to systematically evaluate the efficacy of 8 acupuncture methods in treating central facial palsy after stroke. Due to the different efficacy assessment criteria used in the original studies, this study conducted a stratified analysis of the total effective rate by HB classification and TCM classification. The results showed that, based on the HB standard, acupuncture combined with conventional treatment (AandCT) and acupuncture combined with traditional Chinese medicine (AandTCM) performed the best in improving the total effective rate; based on the TCM standard, fire needle (FN) and scalp acupuncture (SA) had outstanding efficacy. In terms of improving facial function and quality of life (FDI), botulinum toxin type A combined with acupuncture (BTTAandA) had the greatest advantage, and cupping combined with acupuncture (CandA) and Penetrating needling technique combined with moxibustion (PNTandMT) also had significant efficacy. The frequency of acupoint usage statistics showed that Hegu, Dicang, Jiaqu, Sishui, and Xianle are the core acupoints for treating this disease. All combined therapies showed better clinical effects than conventional treatment, and were safe and economically viable. Despite the methodological limitations, such as the difficulty of implementing blinding in acupuncture research and the lack of uniform criteria for assessing efficacy, this study strengthened the reliability of the conclusions through stratified analysis, sensitivity analysis, heterogeneity control, and bias assessment. Future research should focus on: ① conducting high-quality, large-sample, long-term follow-up randomized controlled trials; ② developing standardized acupuncture operation and efficacy assessment systems to promote the integration and standardization of different evaluation systems such as HB classification and TCM standards; ③ using neuroimaging and molecular biology techniques to explain the mechanism of action deeply; ④ verifying its cost-effectiveness in real-world scenarios to promote the standardized application and policy support of acupuncture in stroke rehabilitation.

## Data Availability

The original contributions presented in the study are included in the article/Supplementary material, further inquiries can be directed to the corresponding author.

## Footnotes

1
https://www.crd.york.ac.uk/PROSPERO/view/CRD420261281063
