# Supplementary material for: Comparison of the clinical efficacy of different acupuncture methods in the treatment of post-stroke central facial paralysis—a network meta-analysis based on randomized controlled trials
Source: Front Neurol. 2026 Mar 27;17:1790069. doi: 10.3389/fneur.2026.1790069 (PMC13066168; doi:10.3389/fneur.2026.1790069)
Supplement: Supplementary file 1 [file Table_1.docx]

**1.Retrieval strategies**

PubMed,Embase,Cochrane library,CNKI,VIP,Wanfang,SinoMed

| **Search number** | **Query** |
| --- | --- |
| #1 | Facial Paralysis[MeSH Terms] |
| #2 | Facial Palsy OR Facial Palsies OR Hemifacial Paralysis OR Central Facial Paralyses OR Facial Paresis[Title/Abstract] |
| #3 | #1 OR #2 |
| #4 | Stroke[MeSH Terms] |
| #5 | Stroke OR Cerebrovascular Accident* OR Brain Vascular Accident* OR Apoplexy OR CVA OR apoplectic[Title/Abstract] |
| #6 | #4 OR #5 |
| #7 | Acupuncture OR Acupuncture Therapy OR Acupuncture, Ear OR Moxibustion[MeSH Terms] |
| #8 | Acupuncture OR Moxibustion OR Pharmacopuncture OR Acupotomy OR Acupotomies OR knife needle OR acupotome OR Acusector OR Electroacupuncture OR Acupoint* OR Fu's Subcutaneous Needling OR Moxabustion OR Cupping OR tuina OR massage OR Kinesio Taping OR Chinese  herbal fumigation OR Traditional Chnese medicine steamin[Title/Abstract] |
| #9 | #7 OR #8 |
| #10 | #3 AND #6 AND #9 |

Web of science

| **Search number** | **Query** |
| --- | --- |
| #1 | Facial Palsy OR Facial Palsies OR Hemifacial Paralysis OR Central Facial Paralyses OR Facial Paresis[Title/Abstract] |
| #2 | Stroke OR Cerebrovascular Accident* OR Brain Vascular Accident* OR Apoplexy OR CVA OR apoplectic[Title/Abstract] |
| #3 | Acupuncture OR Moxibustion OR Pharmacopuncture OR Acupotomy OR Acupotomies OR knife needle OR acupotome OR Acusector OR Electroacupuncture OR Acupoint* OR Fu's Subcutaneous Needling OR Moxabustion OR Cupping OR tuina OR massage OR Kinesio Taping OR Chinese  herbal fumigation OR Traditional Chnese medicine steamin[Title/Abstract] |
| #4 | #1 AND #2 AND #3 |

**2.League table**

(1)Efficacy

①HB

| CT | Rood | A | TCM | AandTCM | AandCT | TNandCT | PNTandMT | TN | AandRood |
| --- | --- | --- | --- | --- | --- | --- | --- | --- | --- |
| CT |  |  |  | 0.86 (0.76, 0.96) | 0.84 (0.74, 0.96) | 0.79 (0.57, 1.10) |  |  |  |
| 1.01 (0.67, 1.52) | Rood | 0.95 (0.67, 1.34) |  |  |  |  |  |  | 0.71 (0.54, 0.94) |
| 0.96 (0.77, 1.20) | 0.95 (0.67, 1.34) | A | 0.97 (0.79, 1.20) | 0.89 (0.74, 1.07) |  |  | 0.82 (0.65, 1.04) | 0.81 (0.68, 0.96) | 0.75 (0.58, 0.97) |
| 0.93 (0.75, 1.15) | 0.92 (0.62, 1.38) | 0.97 (0.79, 1.20) | TCM | 0.92 (0.77, 1.09) |  |  |  |  |  |
| 0.86 (0.76, 0.96) | 0.85 (0.57, 1.25) | 0.89 (0.74, 1.07) | 0.92 (0.77, 1.09) | AandTCM |  |  |  |  |  |
| 0.84 (0.74, 0.96) | 0.83 (0.54, 1.28) | 0.87 (0.68, 1.13) | 0.90 (0.70, 1.16) | 0.98 (0.82, 1.18) | AandCT |  |  |  |  |
| 0.79 (0.57, 1.10) | 0.78 (0.46, 1.32) | 0.82 (0.55, 1.22) | 0.85 (0.57, 1.25) | 0.93 (0.65, 1.31) | 0.94 (0.66, 1.34) | TNandCT |  |  |  |
| 0.79 (0.57, 1.09) | 0.78 (0.51, 1.19) | 0.82 (0.65, 1.04) | 0.85 (0.62, 1.16) | 0.92 (0.68, 1.25) | 0.94 (0.66, 1.33) | 1.00 (0.63, 1.58) | PNTandMT |  |  |
| 0.78 (0.59, 1.03) | 0.77 (0.52, 1.13) | 0.81 (0.68, 0.96) | 0.84 (0.64, 1.10) | 0.91 (0.71, 1.17) | 0.93 (0.68, 1.26) | 0.99 (0.64, 1.51) | 0.99 (0.74, 1.32) | TN |  |
| 0.72 (0.52, 1.01) | 0.71 (0.54, 0.94) | 0.75 (0.58, 0.97) | 0.77 (0.56, 1.08) | 0.84 (0.62, 1.16) | 0.86 (0.60, 1.23) | 0.91 (0.57, 1.46) | 0.91 (0.65, 1.29) | 0.93 (0.68, 1.26) | AandRood |

②TCM

| CT | PAandCT | A | BTTA | AandMT | FA | CandA | EARandA | BTTAandA | AandTCM | SA | FN |
| --- | --- | --- | --- | --- | --- | --- | --- | --- | --- | --- | --- |
| CT | 0.81 (0.58, 1.12) | 0.65 (0.42, 1.02) | 0.68 (0.43, 1.07) |  |  |  |  | 0.54 (0.36, 0.81) |  |  |  |
| 0.81 (0.58, 1.12) | PAandCT |  |  |  |  |  |  |  |  |  |  |
| 0.65 (0.42, 1.02) | 0.81 (0.47, 1.40) | A | 1.05 (0.74, 1.47) | 0.90 (0.72, 1.12) | 0.89 (0.69, 1.15) | 0.85 (0.72, 1.02) | 0.82 (0.63, 1.06) | 0.82 (0.62, 1.09) | 0.77 (0.58, 1.03) | 0.77 (0.56, 1.04) | 0.76 (0.59, 0.98) |
| 0.68 (0.43, 1.07) | 0.84 (0.48, 1.47) | 1.05 (0.74, 1.47) | BTTA |  |  |  |  | 0.79 (0.59, 1.05) |  |  |  |
| 0.59 (0.36, 0.96) | 0.73 (0.40, 1.31) | 0.90 (0.72, 1.12) | 0.86 (0.57, 1.29) | AandMT |  |  |  |  |  |  |  |
| 0.58 (0.35, 0.97) | 0.72 (0.39, 1.32) | 0.89 (0.69, 1.15) | 0.85 (0.56, 1.30) | 0.99 (0.71, 1.39) | FA |  |  |  |  |  |  |
| 0.56 (0.35, 0.90) | 0.69 (0.39, 1.23) | 0.85 (0.72, 1.02) | 0.82 (0.56, 1.20) | 0.95 (0.72, 1.26) | 0.96 (0.71, 1.31) | CandA |  |  |  |  |  |
| 0.53 (0.32, 0.89) | 0.66 (0.36, 1.21) | 0.82 (0.63, 1.06) | 0.78 (0.51, 1.20) | 0.91 (0.65, 1.28) | 0.92 (0.64, 1.33) | 0.96 (0.70, 1.31) | EARandA |  |  |  |  |
| 0.54 (0.36, 0.81) | 0.66 (0.39, 1.12) | 0.82 (0.62, 1.09) | 0.79 (0.59, 1.05) | 0.91 (0.64, 1.31) | 0.92 (0.63, 1.35) | 0.96 (0.69, 1.34) | 1.00 (0.68, 1.47) | BTTAandA |  |  |  |
| 0.50 (0.30, 0.85) | 0.62 (0.33, 1.16) | 0.77 (0.58, 1.03) | 0.74 (0.47, 1.15) | 0.86 (0.60, 1.23) | 0.87 (0.59, 1.28) | 0.90 (0.64, 1.26) | 0.94 (0.64, 1.39) | 0.94 (0.63, 1.40) | AandTCM |  |  |
| 0.50 (0.29, 0.86) | 0.62 (0.33, 1.16) | 0.77 (0.56, 1.04) | 0.73 (0.46, 1.16) | 0.85 (0.59, 1.25) | 0.86 (0.58, 1.29) | 0.90 (0.63, 1.28) | 0.94 (0.63, 1.41) | 0.93 (0.62, 1.42) | 1.00 (0.65, 1.52) | SA |  |
| 0.50 (0.30, 0.83) | 0.61 (0.33, 1.13) | 0.76 (0.59, 0.98) | 0.73 (0.47, 1.11) | 0.85 (0.60, 1.19) | 0.86 (0.60, 1.23) | 0.89 (0.65, 1.21) | 0.93 (0.64, 1.34) | 0.93 (0.63, 1.35) | 0.99 (0.67, 1.46) | 0.99 (0.66, 1.48) | FN |

PDI

| CT | A | TCM | EARandA | AandCT | Rood | AandRood | FN | BTTA | AandTCM | TN | AandFN | PNTandMT | TNandCT | CandA | BTTAandA |
| --- | --- | --- | --- | --- | --- | --- | --- | --- | --- | --- | --- | --- | --- | --- | --- |
| CT | -7.50 (-13.21, -1.79) | . | . | -4.60 (-11.66, 2.46) | . | . | . | -9.02 (-14.77, -3.27) | -4.12 ( -9.39, 1.15) | . | . | . | -12.17 (-21.36, -2.98) | . | -15.62 (-21.42, -9.82) |
| -3.58 ( -7.74, 0.59) | A | -0.12 ( -5.30, 5.06) | -0.81 ( -6.13, 4.51) | . | -1.47 ( -6.76, 3.82) | -1.70 ( -7.02, 3.62) | -1.98 ( -7.25, 3.29) | -1.52 ( -7.21, 4.17) | -5.00 ( -8.04, -1.96) | -5.34 (-10.60, -0.08) | . | -7.98 (-15.51, -0.45) | . | -8.04 (-13.27, -2.81) | -8.12 (-13.86, -2.38) |
| -3.55 ( -9.48, 2.38) | 0.03 ( -4.68, 4.73) | TCM | . | . | . | . | . | . | -4.06 ( -9.24, 1.12) | . | . | . | . | . | . |
| -4.39 (-11.15, 2.37) | -0.81 ( -6.13, 4.51) | -0.84 ( -7.94, 6.27) | EARandA | . | . | . | . | . | . | . | . | . | . | . | . |
| -4.60 (-11.66, 2.46) | -1.02 ( -9.22, 7.17) | -1.05 (-10.26, 8.17) | -0.21 ( -9.98, 9.56) | AandCT | . | . | . | . | . | . | . | . | . | . | . |
| -5.05 (-11.78, 1.69) | -1.47 ( -6.76, 3.82) | -1.50 ( -8.58, 5.58) | -0.66 ( -8.17, 6.85) | -0.45 (-10.20, 9.31) | Rood | -0.23 ( -5.55, 5.09) | . | . | . | . | . | . | . | . | . |
| -5.28 (-12.03, 1.48) | -1.70 ( -7.02, 3.62) | -1.73 ( -8.83, 5.37) | -0.89 ( -8.41, 6.63) | -0.68 (-10.45, 9.09) | -0.23 ( -5.55, 5.09) | AandRood | . | . | . | . | . | . | . | . | . |
| -5.56 (-12.27, 1.16) | -1.98 ( -7.25, 3.29) | -2.01 ( -9.07, 5.05) | -1.17 ( -8.66, 6.32) | -0.96 (-10.70, 8.78) | -0.51 ( -7.98, 6.96) | -0.28 ( -7.76, 7.20) | FN | . | . | . | -3.47 ( -7.23, 0.29) | . | . | . | . |
| -7.02 (-12.41, -1.62) | -3.44 ( -8.80, 1.92) | -3.47 (-10.44, 3.51) | -2.63 (-10.18, 4.92) | -2.42 (-11.30, 6.47) | -1.97 ( -9.50, 5.56) | -1.74 ( -9.29, 5.81) | -1.46 ( -8.97, 6.05) | BTTA | . | . | . | . | . | . | -6.60 (-12.37, -0.83) |
| -7.46 (-11.55, -3.38) | -3.89 ( -6.72, -1.05) | -3.91 ( -8.62, 0.79) | -3.08 ( -9.11, 2.95) | -2.86 (-11.02, 5.29) | -2.42 ( -8.42, 3.59) | -2.19 ( -8.21, 3.84) | -1.91 ( -7.89, 4.07) | -0.45 ( -6.15, 5.25) | AandTCM | . | . | . | . | . | . |
| -8.92 (-15.62, -2.21) | -5.34 (-10.60, -0.08) | -5.37 (-12.42, 1.69) | -4.53 (-12.01, 2.95) | -4.32 (-14.05, 5.42) | -3.87 (-11.33, 3.59) | -3.64 (-11.12, 3.84) | -3.36 (-10.80, 4.08) | -1.90 ( -9.41, 5.61) | -1.45 ( -7.43, 4.52) | TN | . | . | . | . | . |
| -9.03 (-16.72, -1.33) | -5.45 (-11.92, 1.02) | -5.48 (-13.47, 2.52) | -4.64 (-13.02, 3.74) | -4.43 (-14.87, 6.01) | -3.98 (-12.34, 4.38) | -3.75 (-12.12, 4.63) | -3.47 ( -7.23, 0.29) | -2.01 (-10.41, 6.39) | -1.56 ( -8.63, 5.50) | -0.11 ( -8.45, 8.23) | AandFN | . | . | . | . |
| -11.56 (-20.16, -2.95) | -7.98 (-15.51, -0.45) | -8.01 (-16.88, 0.87) | -7.17 (-16.39, 2.05) | -6.96 (-18.09, 4.17) | -6.51 (-15.71, 2.69) | -6.28 (-15.50, 2.94) | -6.00 (-15.19, 3.19) | -4.54 (-13.78, 4.70) | -4.09 (-12.14, 3.95) | -2.64 (-11.82, 6.54) | -2.53 (-12.46, 7.40) | PNTandMT | . | . | . |
| -12.17 (-21.36, -2.98) | -8.59 (-18.68, 1.49) | -8.62 (-19.55, 2.31) | -7.78 (-19.19, 3.62) | -7.57 (-19.15, 4.01) | -7.12 (-18.51, 4.27) | -6.89 (-18.29, 4.51) | -6.61 (-17.99, 4.77) | -5.15 (-15.81, 5.50) | -4.71 (-14.76, 5.35) | -3.25 (-14.63, 8.12) | -3.14 (-15.12, 8.84) | -0.61 (-13.20, 11.97) | TNandCT | . | . |
| -11.62 (-18.30, -4.93) | -8.04 (-13.27, -2.81) | -8.07 (-15.10, -1.04) | -7.23 (-14.69, 0.23) | -7.02 (-16.74, 2.70) | -6.57 (-14.01, 0.87) | -6.34 (-13.80, 1.12) | -6.06 (-13.48, 1.36) | -4.60 (-12.09, 2.88) | -4.15 (-10.10, 1.79) | -2.70 (-10.11, 4.71) | -2.59 (-10.91, 5.73) | -0.06 ( -9.23, 9.11) | 0.55 (-10.81, 11.91) | CandA | . |
| -13.62 (-19.06, -8.17) | -10.04 (-15.45, -4.63) | -10.07 (-17.08, -3.05) | -9.23 (-16.82, -1.64) | -9.02 (-17.93, -0.11) | -8.57 (-16.13, -1.00) | -8.34 (-15.92, -0.75) | -8.06 (-15.61, -0.51) | -6.60 (-12.37, -0.83) | -6.15 (-11.90, -0.41) | -4.70 (-12.24, 2.84) | -4.59 (-13.02, 3.84) | -2.06 (-11.33, 7.21) | -1.45 (-12.12, 9.23) | -2.00 ( -9.52, 5.52) | BTTAandA |

**3.Results of the quality assessment of evidence**

| **Intervening measure** | **Comparison** | **Number of studies** | **Within-study bias** | **Reporting bias** | **Indirectness** | **Imprecision** | **Heterogeneity** | **Incoherence** | **Confidence rating** | **Reason(s) for downgrading** |
| --- | --- | --- | --- | --- | --- | --- | --- | --- | --- | --- |
| TER | A:CT | 1 | No concerns | Low risk | No concerns | Some concerns | No concerns | Some concerns | Low | ["Imprecision","Incoherence"] |
|  | AandCT:CT | 2 | No concerns | Low risk | No concerns | No concerns | Some concerns | No concerns | Moderate | ["Heterogeneity"] |
|  | AandTCM:CT | 2 | No concerns | Low risk | No concerns | No concerns | Some concerns | No concerns | Moderate | ["Heterogeneity"] |
|  | BTTA:CT | 1 | No concerns | Low risk | No concerns | Some concerns | No concerns | Some concerns | Low | ["Imprecision","Incoherence"] |
|  | BTTAandA:CT | 1 | No concerns | Low risk | No concerns | No concerns | Some concerns | Major concerns | Very low | ["Heterogeneity","Incoherence"] |
|  | CandA:CT | 1 | No concerns | Low risk | No concerns | Some concerns | Some concerns | No concerns | Low | ["Imprecision","Heterogeneity"] |
|  | CT:EAandCT | 1 | No concerns | Low risk | No concerns | No concerns | Some concerns | No concerns | Moderate | ["Heterogeneity"] |
|  | CT:PAandCT | 1 | No concerns | Low risk | No concerns | Some concerns | No concerns | No concerns | Moderate | ["Imprecision"] |
|  | CT:TNandCT | 1 | No concerns | Low risk | No concerns | Some concerns | No concerns | No concerns | Moderate | ["Imprecision"] |
| PDI | AandCT:CT | 1 | No concerns | Low risk | No concerns | Some concerns | No concerns | No concerns | Moderate | ["Imprecision"] |
|  | AandTCM:CT | 1 | No concerns | Low risk | No concerns | No concerns | Some concerns | No concerns | Moderate | ["Heterogeneity"] |
|  | BTTAandA:CT | 1 | No concerns | Low risk | No concerns | No concerns | Some concerns | No concerns | Moderate | ["Heterogeneity"] |
|  | CT:TNandCT | 1 | No concerns | Low risk | No concerns | Major concerns | No concerns | No concerns | High | ["Imprecision"] |
